# Bacteriophages: what role may they play in life after spinal cord injury?

**DOI:** 10.1038/s41393-021-00636-2

**Published:** 2021-05-07

**Authors:** Lorenz Leitner, Shawna McCallin, Thomas M. Kessler

**Affiliations:** Department of Neuro-Urology, Balgrist University Hospital, University of Zürich, Zürich, Switzerland

**Keywords:** Spinal cord diseases, Urinary tract infection

## Abstract

Bacterial infections are the leading cause of death in people with a spinal cord injury (SCI). Bacteriophages (phages) are viruses that solely infect and kill bacteria. The idea of using phages to treat bacterial infections, i.e., phage therapy, is very promising and potentially allows a more specific and personalized treatment of bacterial infections than antibiotics. While multi-drug resistant infections affect individuals from the general population, alternative therapeutic options are especially warranted in high-risk populations, such as individuals with SCI. However, more clinical data must be collected before phage therapy can be implemented in clinical practice, with numerous possible, subsequent applications.

## Main text

Suffering a spinal cord injury (SCI) is a life-disrupting event with devastating consequences for the individuals and their communities. Advances in primary care, rehabilitation interventions, life-long medical management and institutionalized social support have saved many lives and allow individuals with a SCI to adapt to their new condition and fully participate in social and economic life, at least in high-income countries [1]. However, even apart from the first rehabilitation, people with SCI have more and relatively longer hospital admissions on average than people without SCI, resulting in a higher risk for hospital-acquired illness [2]. Multi-drug resistant (MDR) microbes, potentially hospital-acquired, are a frequent culprit [3]. Despite all advances in modern medicine, the life expectancy in the SCI population is relatively shorter than in the general population, and the leading cause of death in persons surviving the first year post-injury after SCI are infectious diseases, mostly respiratory, urinary tract or skin and bone related [4].

In the context of increasing MDR, current treatment options are failing to provide sufficient therapeutic benefit to individuals with SCI with bacterial infections. Not only does infection severely impact life expectancy and quality of life, but it also increases health care burden and costs. Therefore, the implementation of new treatment strategies and the development of products with reliable performance in daily clinical practice is indispensable.

## Phages and therapeutic application

**Fig. 1:**
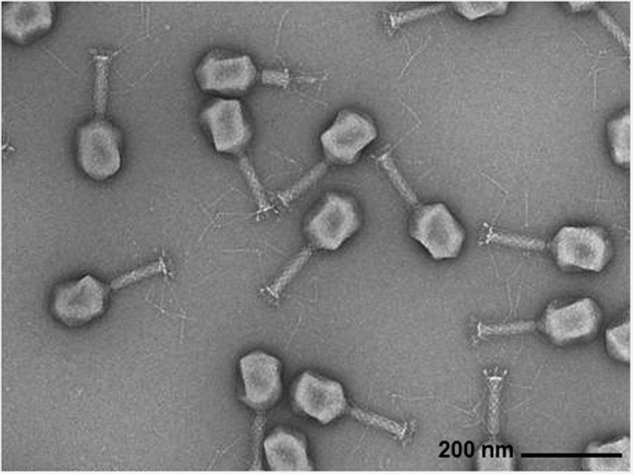
Transmission electron microscopy (TEM) image of bacteriophages (phages) produced to treat a patient with a multi-resistant *Enterobacter cloacae* urinary tract infection. The phages are composed of a capsid (containing the genome) connected to a contractile tail with protruding tail fibers which mediate interaction with bacterial surface receptors. Phages are highly host specific, meaning a given phage is capable of infecting only a narrow host range of bacteria (typically down to the species level). TEM image was kindly provided by Matthew Dunne, Ph.D., and Jiemin Du, M.Sc., from the Institute of Food, Nutrition and Health, ETH Zürich, Zürich, Switzerland.

Bacteriophages [5, 6] (phages), from the Greek word *βακτήριoν* (baktérion) and *ϕαγεῖν* (phageín) (meaning: “bacteria eaters”), have the potential to complement and optimize current treatment strategies in infectious diseases and other medical fields (Fig. 1). Phages are naturally-existing viruses that specifically kill bacteria, including MDR strains, while eukaryotic cells are not infected. Unlike antibiotics, phages are highly specific to the bacterial species, or even to the bacterial strain.

The idea of using phages to treat bacterial infections, i.e., phage therapy, is not new. In 1919, Félix d’ Hérelle conducted the first initial experiments with phages to cure bacterial infections in humans and successfully treated several children at the Hospital des Enfants Malades in Paris [6]. However, the discovery of penicillin a decade later (1928) deterred the interest for phages in the Western world. At present, phage therapy is a well-accepted and registered therapy in post-Soviet countries like Georgia, Ukraine, and Russia. However, amidst the growing concerns of antimicrobial resistance, the use of phages as a target therapy against bacterial pathogens has gained a renewed interest worldwide. While reports of successfully applied phage therapies for different medical indications have been published and case reports demonstrate successful treatment outcomes for some patients, there is still a lack of high-quality evidence to support the use of this treatment strategy. Nevertheless, more and more academic and industrial consortia are entering the field, aiming to implement phage and phage-related therapeutic pathways in routine clinical practice.

## Phage therapy in the context of SCI

Several treatment strategies and recent advances in phage therapy could be of specific interest for the SCI population, such as urinary tract infections (UTIs), as well as respiratory and skin or bone infections. While none of the currently available literature or case reports for phage therapy involve the treatment of individuals with SCI, their experiences are relevant and illustrate future potential applications for the SCI population.

Virtually every individual with SCI suffers from neurogenic lower urinary tract dysfunction, a condition leading to recurrent UTIs in many patients. UTI is associated with a considerable morbidity and mortality and substantially affects the quality of life. In vitro results of a study performed by our group found high lytic activity of a Georgian, commercially-available and licensed phage product against most common bacterial strains detected in UTIs from individuals with SCI [7]. A subsequent, randomized controlled trial (RCT) [8] comparing intravesical instillations of either a phage or a placebo versus standard-of-care (SOC) antibiotic treatment showed that intravesical phage therapy was non-inferior to the SOC, but not superior to placebo bladder irrigation in terms of efficacy in treating UTIs in patients undergoing transurethral resection of the prostate. Moreover, the phage safety profile seemed to be favorable compared to the other treatment options. However, the study did not include individuals with SCI or patients performing intermittent self-catheterization, which would be more relevant populations for understanding the value of phage therapy for SCI. In a step to address this, a RCT in this populations, using phages specifically engineered to treat catheter-associated UTIs, will be starting soon (http://p3.snf.ch/project-189957). Results from other upcoming clinical trials (clinicaltrials.gov: NCT04287478, NCT04191148) focusing on UTI will further enhance our understanding.

Another main focus for phage therapy is the treatment of respiratory tract infections particularly for patients with cystic fibrosis (CF), who are at an increased risk of developing MDR bacterial infections. After several emergency treatments with phages for CF patients, two clinical trials are starting for the treatment of *Pseudomonas aeruginosa* infections (clinicaltrials.gov: NCT04684641, NCT04596319). This is worth mentioning, as even if CF or *P. aeruginosa* lung infections are not directly SCI related, insights in terms of potential strategies for phage delivery (e.g., topical/inhalation, oral, intravenous), to optimize therapeutic effects by degradation of biofilms and enhancing bacteria phage interactions should not be underestimated for all kind of bacterial infections. Additionally, the Food and Drug Administration (FDA) recently authorized the use of phage for the treatment of bacterial pneumonia co-infections in COVID-19 patients (clinicaltrials.gov: NCT04636554).

Individuals with SCI are frequently afflicted by pressure sores often aggravated by superinfections and potentially causing osteomyelitis. Phages provide an interesting platform in treating osteomyelitis and peri-prosthetic joint infection (PJI) [9], resulting often in positive clinical outcomes. Most case reports have entailed surgical procedures to apply phage directly to the site of infection, as well as debridement of the infection site or prosthesis exchange, but intravenous application has been more recently reported. Published reports for skin indications are less frequent: for one patient who underwent amputation of the lower limbs, septic episodes caused by a polymicrobial infected pressure sore were controlled with phage therapy [10]. The ability of phages to both degrade biofilms and eliminate bacteria represents a great potential for the treatment of skin and bone infections in individuals with SCI. While preclinical studies and case reports have shown promising results, clinical trials are still needed to evaluate phage therapy more vigorously. The FDA has recently granted orphan drug designation of phage therapy for the treatment of osteomyelitis and PJI, which indicates that trials are soon to follow [9].

## Phage therapy: next steps and other areas of application

Why has the Western world not yet implemented phage therapy, despite the long history, the high potential, and the urgent need? There are several reasons. Most of the time, antibiotic therapy has provided, and still does provide, excellent results for patients. Hence, the interest to implement new therapeutic strategies to treat bacterial infections have long-since been of low priority, especially for strategies with complex regulatory pathways and low potential for financial returns, such as phage therapy. At present, phage therapy is not authorized outside of clinical studies in most countries, but it is occasionally used as an experimental treatment in emergency cases (i.e. compassionate use). Considering that phages are host-specific and target only a subset of bacterial strains, a broad arsenal of different phages is required to find the right phage for each patient. This has posed considerable problems regarding regulations and manufacturing compared to small-molecule development. In this perspective, recent approval by the FDA for a phage collection for personalized therapies (PhageBank^TM^ by the Biological Defense Research Directorate of the U.S. Naval Medical Research Center (NMRC), which will be used in the clinical trial NCT04287478), as well as the Belgian regulatory approach (Belgium Phage Active Pharmaceutical Ingredient monograph (version 1.0) for magistral phage preparation: http://www.mdpi.com/1999-4915/10/2/64/s1) of allowing custom-made phage medicines for individual patients, provide light at the end of the tunnel and open the gates for phage therapy to progress into clinical medicine.

Advances in molecular biology and bioengineering also extend the field for even more phage-related medical applications. On one hand, phages can be engineered to enhance their host range or to augment their antimicrobial activity. Another exciting strategy is to include payload genes in a phage genome so that host bacteria would produce and dispense specific proteins (e.g., with immune-modulatory or anti-inflammatory effects) at the site of infection. The ability of phage to cross the blood-brain-barrier [11] could further allow their use as a drug delivery vectors to the central nervous system, an idea of specific interest in patients with acute SCI.

## Conclusions

Phage therapy is very promising and potentially allows a more specific and personalized treatment of bacterial infections. While MDR infections affect individuals from the general population, alternative therapeutic options are especially warranted in high-risk populations, such as individuals with SCI. However, which phages to use and how exactly to apply them (concentration, administration route, duration, posology) is challenging to define. Only once more clinical data has been obtained will it be possible to implement phage therapy in clinical practice, and thus open the door for numerous future applications.
